# Genotype Differences and Hydroxyurea Utilization Among Adults With Moderate to Severe Sickle Cell Disease

**DOI:** 10.1002/phar.70099

**Published:** 2026-01-14

**Authors:** Siang‐Hao Cheng, Enrico M. Novelli, Hyeun Ah Kang, Terri V. Newman, Kangho Suh

**Affiliations:** ^1^ Department of Pharmacy & Therapeutics University of Pittsburgh School of Pharmacy Pittsburgh Pennsylvania USA; ^2^ University of Pittsburgh School of Medicine Pittsburgh Pennsylvania USA; ^3^ The University of Texas at Austin College of Pharmacy Health Outcomes Division Austin Texas USA

**Keywords:** anemia, drug utilization, hemoglobin SC disease, hydroxyurea, sickle cell, vaso‐occlusive crises

## Abstract

**Backgrounds:**

Hydroxyurea (HU) remains underutilized in adults with sickle cell disease (SCD) despite proven benefits. Current HU guidelines primarily target sickle cell anemia (SCA), overlooking other genotypes.

**Objectives:**

This study examined HU utilization patterns across genotypes among adults considered to have moderate to severe SCD manifestations by the 2014 National Heart, Lung, and Blood Institute (NHLBI) guideline criteria and identified factors associated with early HU use.

**Methods:**

This retrospective cohort study analyzed electronic health records from the University of Pittsburgh Medical Center (2014–2024) of adults with SCD experiencing three or more vaso‐occlusive crises (VOCs) within 12 months. HU utilization rates, stratified by genotype, were assessed at 30‐, 90‐, 180‐, and 365‐day intervals after the third VOC episode (*index date*). Multivariable logistic regression was used to identify factors associated with HU use within 90 days post‐index.

**Results:**

Among 411 adults with moderate to severe SCD (≥ 3 VOCs within a year), with a mean age of 42.4 ± 17.9 years and 61.3% female, only 19.5% received HU within 90 days post‐index. Although 42.8% of SCA patients received HU within 1 year, only 8.0% of non‐SCA patients received the treatment. The SCA genotype was the strongest predictor of HU use (odds ratio [OR] = 4.5, 95% confidence interval [CI]: 2.4–8.7), followed by pulmonary complications. Additional barriers included older age.

**Conclusion:**

Despite guideline recommendations since 2014, HU remains underutilized. Non‐SCA patients meeting the severity threshold for HU use are consistently undertreated, highlighting an urgent need for studies establishing HU safety and efficacy in non‐SCA genotypes. Future studies should also address age barriers to optimize HU use.

## 1 Introduction

The landscape of sickle cell disease (SCD) has changed drastically in recent decades. Although once primarily considered a pediatric disease, improved medical care in high‐income countries now enables nearly 95% of children with SCD to survive into adulthood [1]. This success has transformed SCD into a chronic condition requiring lifelong management [2], and is still plagued by numerous challenges including barriers to care [3], decreased quality of life [4], and huge economic burden on patients and the health care system [5, 6].

Hydroxyurea (HU) remains the most important disease‐modifying therapy for SCD with the longest history of development and strong evidence demonstrating its efficacy in reducing vaso‐occlusive crises (VOCs), hospitalizations, and mortality rates in both pediatric and adult populations [7, 8, 9]. Consequently, the 2014 National Heart, Lung, and Blood Institute (NHLBI) guidelines recommend HU for adult patients with sickle cell anemia (SCA), defined as patients with HbSS or HbSβ0 thalassemia genotypes, who experience three or more moderate to severe VOCs within a 12‐month period [8, 10].

Despite these recommendations and proven benefits, HU remains underutilized in the United States, particularly among adults [11]. A 2015 claims data study revealed that fewer than 25% of eligible adult patients with SCA received HU within 1 year of meeting NHLBI guideline criteria [11]. Similarly, a Florida study examining HU utilization between 2012 and 2017 found that despite a modest increase following the 2014 NHLBI guidelines (from 12.7% to 17.4%), HU utilization remained low, particularly among older individuals [12]. A recent study further confirms persistently low HU utilization among the entire SCD population in the United States, with rates remaining below 25% by 2021 [13].

Although HU utilization patterns are well‐described, existing literature evaluating HU use has limitations. Current NHLBI guidelines primarily target patients with SCA, despite growing evidence that HU therapy may provide clinical benefits to non‐SCA genotypes (e.g., HbSC and HbSβ+ thalassemia) [14, 15], which together with HbSS/HbSβ0 thalassemia represent approximately 99% of all SCD cases [16]. Although HbSC (representing most non‐SCA cases) disease accounts for approximately 30% of SCD cases in the United States [17], this genotype receives insufficient attention in current guidelines despite evidence that it is no longer considered mild, as patients can present with severe clinical outcomes [18, 19, 20, 21]. The underrepresentation of non‐SCA genotypes in HU research creates a critical knowledge gap, especially since patients with moderate to severe VOCs across all SCD genotypes may become candidates for HU therapy according to current clinical evidence. This gap is further widened by the lack of updated comprehensive studies examining guideline adherence among adult patients with SCD for nearly a decade since a 2015 investigation [11]. In today's era of accelerated scientific advancement in SCD research, substantial evidence has emerged suggesting that HU's therapeutic value could extend to adult patients across all genotypic variants, yet there remains limited research exploring current utilization rates and factors influencing HU use in adults with moderate to severe SCD defined by NHLBI guidelines.

To develop a more comprehensive understanding of HU use in the modern SCD treatment landscape, our first objective was to assess patterns of HU utilization across different genotypes among adults experiencing three or more moderate to severe VOCs within 30‐, 90‐, 180‐, and 365‐day intervals following an *index* VOC episode. Our second objective investigated patient characteristics associated with early HU utilization (within 90 days post‐index) in both SCA and non‐SCA patients with moderate to severe diseases, populations for whom current evidence regarding treatment patterns remains limited.

## 2 Methods

### 2.1 Data Source

This retrospective cohort study analyzed electronic health record (EHR) data from the University of Pittsburgh Medical Center (UPMC) health system from January 1, 2014, to December 31, 2024. UPMC operates as one of 14 designated Pennsylvania centers providing comprehensive hemoglobinopathy care through its specialized adult SCD program that also captures internal prescriptions and pharmacy fills from external providers for enrolled patients. The EHR also encompasses clinical, administrative, and financial datasets across the continuum of care [22]. As the primary regional referral center for SCD care, this dataset represents the broader western Pennsylvania SCD population. The University of Pittsburgh's institutional review board approved this study with an exemption from informed consent requirements based on the use of deidentified patient data.

### 2.2 Study Population and Definitions

We identified adult patients (≥ 18 years) with SCD using a validated algorithm that requires either two outpatient visits ≥ 30 days apart or one hospitalization with an SCD diagnosis (*International Classification of Diseases, Ninth Revision* [*ICD‐9*]: 282.4x, 282.6x; *International Statistical Classification of Diseases and Related Health Problems, Tenth Revision* [*ICD‐10*]: D57.x, excluding sickle cell trait) [23].

VOC episodes were identified using ICD‐9/ICD‐10 codes for SCD with crisis, acute chest syndrome (ACS), splenic sequestration, priapism, and supplementary pain‐related diagnoses commonly associated with SCD crises (abdominal/pelvic, chest, joint, limb, and back pain) [11, 24]. These VOC episodes were considered moderate to severe as they required medical care with physician visits, including inpatient, outpatient, or emergency department visits. VOC‐related encounters occurring within 7 days of each other were considered part of the same episode [25]. We excluded patients with malignancies that may require HU treatment and those who had undergone hematopoietic stem cell transplantation (HSCT) [25]. Detailed codes for SCD, VOC, and inclusion–exclusion criteria are listed in Tables S1–S3.

### 2.3 Study Design

To identify patients eligible for HU based on the NHLBI guideline, we applied a sliding window approach across each patient's records to identify the earliest 12‐month period in which three or more moderate to severe VOC‐related outpatient, ED, or inpatient visits occurred, with the third VOC occurring on or after October 1, 2014 (following publication of the updated NHLBI guidelines in September 2014). Only the first qualifying window per patient was retained, and the date of the third VOC in that window was defined as the *index date*.

We established an analytic cohort requiring evidence of ongoing health care system engagement through one or more documented health care activities (including clinical visits, laboratory tests, diagnostic procedures, or pharmacy dispensing records) in both the 180 days pre‐index and 365 days post‐index observation periods. This approach follows established practices for EHR‐based studies that use health care activities as proxies for continuous data availability when enrollment information is unavailable [26, 27]. The 180‐day pre‐index period was used to measure baseline characteristics, while the 365‐day post‐index period ensured sufficient follow‐up time to capture HU utilization rates at multiple time intervals (30‐, 90‐, 180‐, and 365‐day post‐index). Exclusion criteria were applied after confirming these health care engagement criteria.

For both objectives, HU utilization was defined as one or more HU dispensing event or documented inpatient HU administration record within the specified time periods. For the second objective, early HU use was defined as HU utilization within 90 days post‐index. The 90‐day window was selected to allow sufficient time for clinical decision‐making following the index VOC episode while capturing early treatment initiation. To assess temporal patterns in early HU use following the NHLBI guideline publication, we also examined HU utilization rates by index year across the study period.

We allowed a brief implementation period following the September 2014 NHLBI guideline release, with our observation period beginning October 1, 2014. Our analysis showed no significant change in HU utilization rates during this period.

### 2.4 Covariates

We collected variables for baseline comparisons and to evaluate factors associated with early HU use. These variables included age, sex, genotype (SCA [HbSS/Sβ^0^] versus non‐SCA), history of transfusion, opioid use, prior HU use, and VOC episode counts. SCD‐related complications included stroke (ischemic/hemorrhagic)/transient ischemic attack (TIA), multi‐organ failure (MOF), avascular necrosis (AVN), renal disease (chronic kidney disease [CKD], acute or chronic renal failure, and acute and chronic glomerulonephritis), pulmonary complications (pneumonia, upper respiratory tract infection [URTI], pulmonary embolism [PE], and pulmonary hypertension [PH]), thrombosis, and leg ulcers. Non‐SCD‐related comorbidities included chronic obstructive pulmonary disease (COPD)/asthma, heart failure (HF)/coronary artery disease (CAD), cardiometabolic conditions (diabetes [DM], hypertension [HTN], hyperlipidemia [HLD]), and psychiatric disorders (anxiety/depression). ACS was excluded from pulmonary complications and included in VOC counts to avoid double‐counting in the logistic regression. SCA was identified using a validated algorithm by first scanning the most common SCD type based on ICD codes per patient, then the second most common SCD type, followed by assigning patients with HbS ≥ 80% or with Transcranial Doppler (TCD) ultrasound records to SCA [24]. The algorithm for identifying SCA and diagnosis codes for covariates are in Tables S2 and S3, respectively.

### 2.5 Statistical Analysis

For objective 1, HU utilization rates were calculated at each time point by genotype. Baseline characteristics were compared between SCA and non‐SCA patients using *t*‐tests (or Mann–Whitney *U* tests for non‐normally distributed data) for continuous variables and chi‐square tests (or Fisher's exact test when expected cell counts were ≤ 5) for categorical variables. For objective 2, baseline characteristics were compared between early HU users versus non‐early HU users using the same methods. Multivariable logistic regression models were used to identify independent predictors of early HU use (within 90 days post‐index), followed by three sensitivity analyses. For all models, we included baseline demographic variables and clinical variables with prevalence ≥ 5% to ensure sufficient events for stable model estimation while maintaining clinical relevance to treatment decisions [10, 28]. To develop our primary (reduced) model and ensure reliable coefficient estimates, variables with quasi‐complete separation or fewer than 10 HU users in any category were excluded to prevent unreliable coefficient estimates or disproportionately high odds ratios that obscure meaningful associations of other variables with the outcome in logistic regression. Specifically, compared with the full model, the primary model excluded prior HU use, renal disease, HF/CAD, and DM/HTN/HLD due to these constraints. The three sensitivity analyses included: (i) a full model using Firth's logistic regression with all baseline variables (prevalence ≥ 5%), (ii) analysis restricted to SCA patients (*n* = 187), and (iii) analysis using an 180‐day treatment window. Analyses 2 and 3 used the reduced model variable set. Statistical analyses were performed using SAS 9.4, with *p* < 0.05 considered significant.

## 3 Results

### 3.1 Objective 1: HU Utilization Patterns and Genotype Differences

#### 3.1.1 Baseline Characteristics by SCD Genotype

**FIGURE 1:**
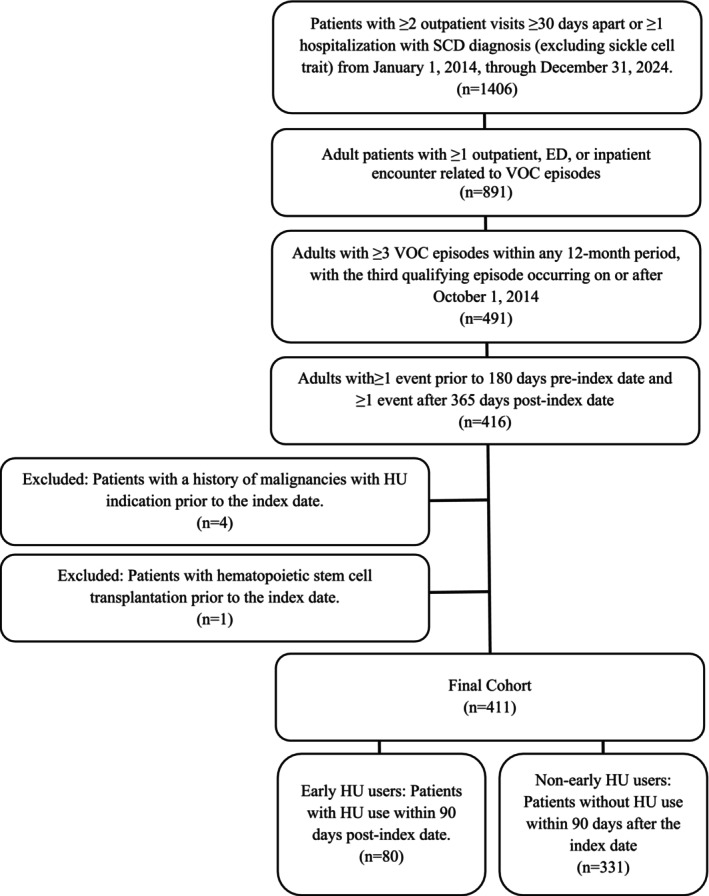
Sample selection process. The index date was defined as the date of the third VOC episode within the earliest 12‐month period with ≥ 3 VOCs, with the third qualifying episode occurring on or after October 1, 2014. ED, emergency department; HU, hydroxyurea; SCD, sickle cell disease; VOC, vaso‐occlusive crisis.

The sample selection process is shown in Figure 1. Among all SCD patients identified during the study period (*n* = 1406), 411 patients had three or more moderate to severe VOC episodes within 12 months and met all inclusion and exclusion criteria, with *index* years spanning 2014–2023 and predominantly representing 2014–2021 (94.4%). As shown in Table 1, the overall cohort had a mean age of 42.4 ± 17.9 years and was predominantly female (61.3%). Of the 411 patients, 187 (45.5%) were classified as SCA and 224 (54.5%) as non‐SCA.

**TABLE 1:** Baseline characteristics and differences between adults with sickle cell disease: SCA versus non‐SCA and those who received hydroxyurea within 90 days after third VOC episode within 1 year (early HU user) versus those who did not (non‐early HU user).

	All (*N* = 411)	Objective 1			Objective 2		
		SCA (*N* = 187)	Non‐SCA (*N* = 224)	*p*	Early HU users (*N* = 80)	Non‐early HU users (*N* = 331)	*p*
		*N* (%)			*N* (%)		
Characteristic							
Age at the index date, years							
Mean ± SD	42.36 ± 17.92	38.04 ± 17.93	45.97 ± 17.12	**< 0.0001** ^a^	29.94 ± 11.27	45.36 ± 17.95	**< 0.0001** ^a^
Median (Q1, Q3)	40.30 (26.1, 56.0)	33.6 (23.1, 47.6)	46.7 (29.85, 59.1)		26.15 (20.55, 36.5)	45.2 (29.5, 58.9)	
Age groups, years							
18–21	48 (11.68)	33 (17.65)	15 (6.70)	**< 0.0001** ^b^	20 (25.00)	28 (8.46)	**< 0.0001** ^b^
22–30	85 (20.68)	44 (23.53)	41 (18.30)		28 (35.00)	57 (17.22)	
31–40	72 (17.52)	38 (20.32)	34 (15.18)		17 (21.25)	55 (16.62)	
41+	206 (50.12)	72 (38.50)	134 (59.82)		15 (18.75)	191 (57.70)	
Sex							
Female	252 (61.31)	100 (53.48)	152 (67.86)	**< 0.01** ^b^	42 (52.50)	210 (63.44)	0.07^b^
Male	159 (38.69)	87 (46.52)	72 (32.14)		38 (47.50)	121 (36.56)	
SCA					64 (80.00)	123 (37.16)	**< 0.0001** ^b^
History of transfusion	24 (5.84)	21 (11.23)	3 (1.34)	**< 0.0001** ^b^	10 (12.50)	14 (4.23)	**< 0.01** ^b^
Opioid use	153 (37.23)	72 (38.50)	81 (36.16)	0.62^b^	33 (41.25)	120 (36.25)	0.41^b^
Prior HU use	73 (17.76)	61 (32.62)	12 (5.36)	**< 0.0001** ^b^	59 (73.75)	14 (4.23)	**< 0.0001** ^b^
VOC episode counts							
Mean ± SD	1.71 ± 1.69	1.96 ± 2.20	1.50 ± 1.08	0.13^a^	2.21 ± 2.83	1.59 ± 1.25	0.49^c^
Median (Q1, Q3)	2 (1, 2)	2 (1, 2)	2 (1, 2)		2 (1, 2)	2 (1, 2)	
SCD‐related complications and comorbidities							
Stroke	16 (3.89)	13 (6.95)	3 (1.34)	**< 0.01** ^c^	2 (2.50)	14 (4.23)	0.47^c^
AVN	7 (1.70)	3 (1.60)	4 (1.79)	0.89^b^	5 (6.25)	2 (0.60)	**< 0.001** ^b^
Renal disease	26 (6.33)	13 (6.95)	13 (5.80)	0.63^b^	7 (8.75)	19 (5.74)	0.32^b^
Pulmonary complications	64 (15.57)	40 (21.39)	24 (10.71)	**< 0.01** ^b^	22 (27.50)	42 (12.69)	**< 0.01** ^b^
Thrombosis	16 (3.89)	9 (4.81)	7 (3.13)	0.38^b^	2 (2.50)	14 (4.23)	0.75^c^
Leg ulcers	8 (1.95)	5 (2.67)	3 (1.34)	0.48^c^	5 (6.25)	3 (0.91)	**< 0.01** ^c^
Other complications and comorbidities							
COPD/asthma	78 (18.98)	37 (19.69)	41 (18.30)	0.70^b^	16 (20.00)	62 (18.73)	0.80^b^
HF/CAD	27 (6.57)	17 (9.09)	10 (4.46)	0.06^b^	3 (3.75)	24 (7.25)	0.26^b^
DM/HTN/HLD	116 (28.22)	37 (19.79)	79 (35.27)	**< 0.001** ^b^	7 (8.75)	109 (32.93)	**< 0.0001** ^b^
Anxiety/depression	98 (23.84)	32 (17.11)	66 (29.46)	**< 0.01** ^b^	12 (15.00)	86 (25.98)	**0.04** ^b^

*Note:* No patients experienced multiorgan failure during the study period. Bolded *p*‐values indicate statistical significance (*p* < 0.05).

Abbreviations: AVN, avascular necrosis; CAD, coronary artery disease; CKD, chronic kidney disease; COPD, chronic obstructive pulmonary disease; DM, diabetes mellitus; HF, heart failure; HLD, hyperlipidemia; HTN, hypertension; HU, hydroxyurea; PE, pulmonary embolism; PH, pulmonary hypertension; SCA, sickle cell anemia; SCD, sickle cell disease; TIA, transient ischemic attack; URTI, upper respiratory tract infection; VOC, vaso‐occlusive crisis.

^a^ Mann–Whitney *U* test.

^b^ Chi‐square test.

^c^ Fisher's exact test.

SCA patients were younger than non‐SCA patients (38.0 ± 17.9 vs. 46.0 ± 17.1 years, *p* < 0.001) and less likely to be females (53.5% vs. 67.9%, *p* < 0.01). Regarding clinical characteristics, SCA patients demonstrated markers of more severe disease, including higher rates of transfusion history, stroke, and pulmonary complications (all *p* < 0.01). Additionally, a higher proportion of SCA patients were already on HU at baseline compared with non‐SCA patients (32.6% vs. 5.4%, *p* < 0.0001). In contrast, non‐SCA patients had higher rates of cardiometabolic conditions (DM/HTN/HLD), as well as higher rates of anxiety/depression compared with SCA patients (all *p* < 0.01).

#### 3.1.2 HU Utilization According to Guideline Recommendation Over Time

**FIGURE 2:**
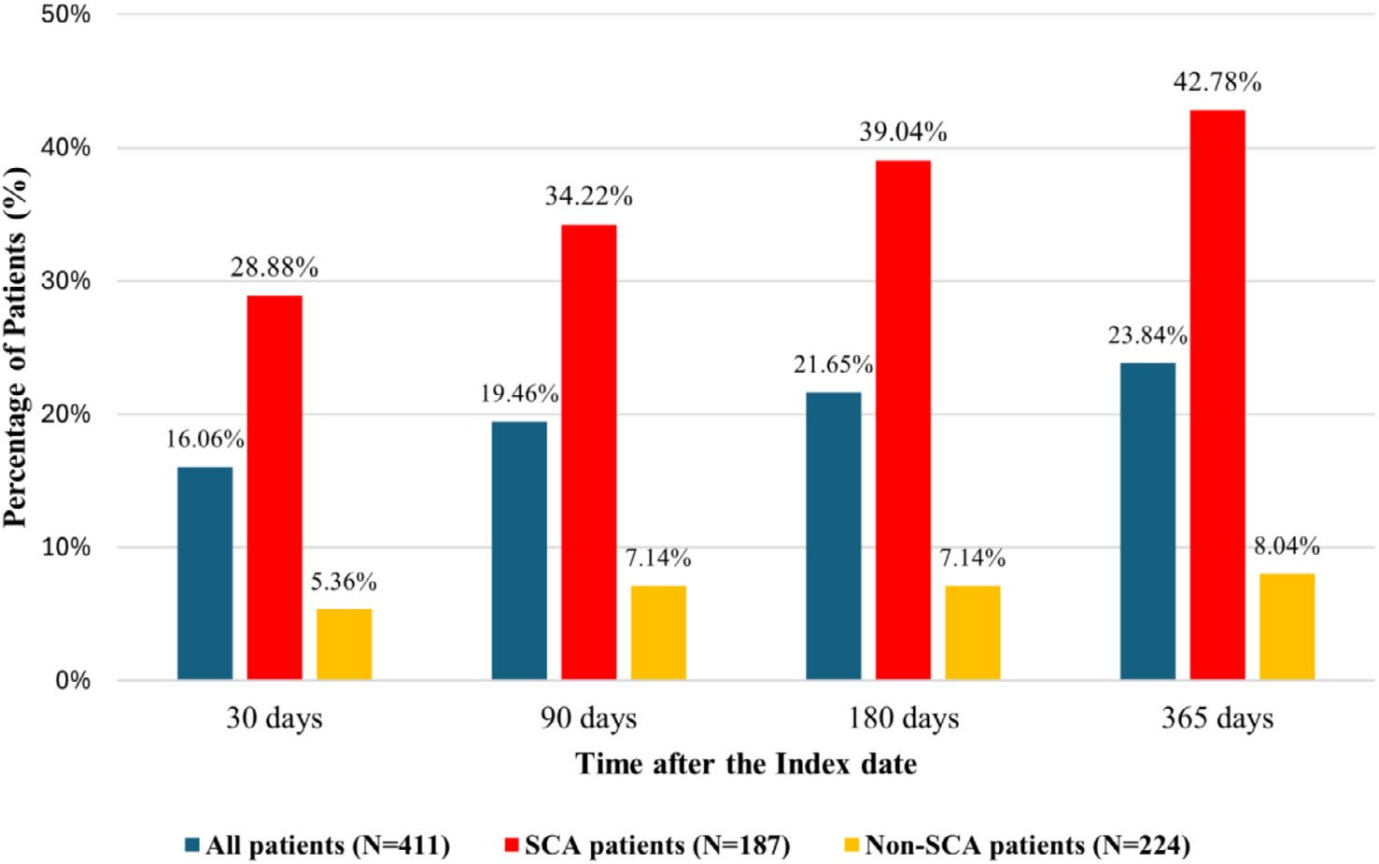
Hydroxyurea utilization rates in adult patients with ≥ 3 VOCs within a year, stratified by sickle cell disease genotype. SCA, sickle cell anemia. SCA includes HbSS and HbSβ^0^‐thalassemia genotypes. Non‐SCA genotypes include HbSC, HbSβ^+^‐thalassemia, and other variants.

As shown in Figure 2, among 411 patients, there was a gradual increase in HU use over time following the *index* date, with 66 (16.1%) patients receiving HU within 30 days post‐index, rising to 80 (19.5%) within 90 days, 89 (21.7%) within 180 days, and 98 (23.8%) within 365 days post‐index.

Among patients with SCA (*n* = 187), utilization rates were higher, with 54 (28.9%) patients having HU within 30 days post‐index, increasing to 64 (34.2%) within 90 days, 73 (39.0%) within 180 days, and 80 (42.8%) within 365 days post‐index. In contrast, patients with non‐SCA genotypes (*n* = 224) had lower utilization rates, with 12 (5.4%) patients having HU within 30 days post‐index, 16 (7.1%) within both 90 and 180 days, and 18 (8.0%) within 365 days post‐index.

### 3.2 Objective 2: Factors Associated With Early HU Use

#### 3.2.1 Baseline Characteristics by Early HU Use

The trends observed between early HU users and non‐early users closely mirror those seen between SCA and non‐SCA patients (Table 1). Of the 411 patients, 80 (19.5%) received HU within 90 days post‐index (early HU users). Early HU utilization rates ranged from 8.6% to 38.5% across study years (Table S4). Early HU users were younger than non‐early users (29.9 ± 11.3 vs. 45.4 ± 18.0 years; *p* < 0.001) and had a lower proportion of females (52.5% vs. 63.4%; *p* = 0.07). Genotype distribution differed, with 80.0% of early HU users having SCA compared with 37.2% of non‐early HU users (*p* < 0.0001).

Early HU users demonstrated markers of more severe disease, including higher rates of transfusion history, avascular necrosis, pulmonary complications, and leg ulcers (all *p* < 0.01). In contrast, non‐early HU users had higher rates of cardiometabolic conditions and anxiety/depression (all *p* < 0.05) compared with early HU users. A sensitivity analysis using an 180‐day post‐index window to define early HU users (89 patients, 21.7%) showed similar patterns in baseline characteristics between the two groups (Table S5).

#### 3.2.2 Multivariable Analysis of Factors Associated With Early HU Use

The *primary* (reduced) model (area under the curve [AUC] = 0.82) identified several independent factors associated with early HU use (Table 2). SCA patients had higher odds of early HU use compared with non‐SCA patients (odds ratio [OR] = 4.5, 95% confidence interval [CI]: 2.4–8.7). Age was strongly associated with HU use, with the likelihood of early HU use decreasing with increasing age. Compared with patients aged 18–21 years, older age groups, especially those aged 40+ years, had significantly lower odds of early HU use. Patients with pulmonary complications were also associated with increased odds (OR = 2.2, 95% CI: 1.1–4.5) for early HU use.

**TABLE 2:** Adjusted odds ratios with 95% confidence intervals for early hydroxyurea utilization.

	Primary model (reduced model)			
	OR	LCI	UCI	*p*
Age 22–30 vs. 18–21 years	0.71	0.31	1.60	**0.03**
Age 31–40 vs. 18–21 years	0.39	0.16	0.94	0.77
Age 40+ vs. 18–21 years	0.11	0.05	0.28	**< 0.0001**
Male vs. female	1.14	0.63	2.05	0.67
SCA vs. non‐SCA	4.54	2.38	8.65	**< 0.0001**
History of transfusion	1.20	0.43	3.35	0.72
Opioid use	1.83	0.99	3.38	0.05
VOC episode counts	1.04	0.89	1.22	0.66
Pulmonary complications	2.23	1.10	4.51	**0.03**
COPD/asthma	1.03	0.48	2.19	0.94
Anxiety/depression	0.73	0.33	1.61	0.43

*Note:* Bolded *p*‐values indicate statistical significance (*p* < 0.05).

Abbreviations: COPD, chronic obstructive pulmonary disease; LCI, lower confidence interval; OR, odds ratio; SCA, sickle cell anemia; UCI, upper confidence interval; VOC, vaso‐occlusive crisis.

In the full model (AUC = 0.93), prior HU use emerged as the strongest predictor (OR = 41.6, 95% CI: 17.2–100.7) for early HU use. This attenuated the associations observed for genotype, age, and pulmonary complications in the primary model. Although no longer statistically significant, these variables maintained similar directional trends. Results were robust across other sensitivity analyses, with consistent patterns observed despite some variations in effect magnitude (Table S6).

## 4 Discussion

This is the first study describing HU utilization rates based on 2014 NHLBI guideline recommendations in centers with established adult SCD programs while including non‐SCA genotypes. We applied a validated algorithm to identify SCD genotypes and conducted multiple sensitivity analyses to enhance the robustness of our findings [24]. Among 1406 patients with SCD identified during the study period, we found 411 (both SCA and non‐SCA genotypes) who met the 2014 guideline severity threshold for HU recommendation (≥ 3 moderate to severe VOCs within 12 months). Among patients meeting this severity threshold, 42.8% of SCA patients and only 8.0% of non‐SCA patients received HU within 1 year. Multivariable analysis revealed that SCA genotype, younger age (18–21 years), and pulmonary complications are associated with early HU use. This contemporary, rigorously designed analysis of HU utilization patterns in an adult cohort underscores the low utilization rates among non‐SCA patients with recurrent VOCs, who remain underserved despite potentially benefiting from HU treatment.

We discovered that HU utilization rates among SCA patients with ≥ 3 VOC episodes in our single‐center cohort were higher (42.8% within 12 months) compared with previous studies. A 2015 study, which evaluated the guideline‐recommended population using national claims data, reported only 22.7% utilization at 12 months [11]. Similarly, other studies have documented consistently low HU utilization rates. The Florida study using the statewide clinical database found less than 20.0% HU use in adults from 2012 to 2017, and a more recent analysis (2017–2021 using Optum data) reported less than 25.0% utilization [12, 13]. Importantly, these latter studies did not specifically focus on adults with frequent VOCs as ours did. Our study provides the most current utilization rates while identifying factors associated with early HU use, particularly in settings with established regional SCD adult programs. The improved utilization rates we observed may reflect methodological differences, data source variations, or genuine clinical progress through specialized SCD programs and enhanced physician prescribing practices. Nevertheless, nearly 58.0% of eligible SCA patients still did not receive HU within 1 year, demonstrating that utilization remains suboptimal and requires improvement. HU underutilization would likely be substantially greater in populations without access to specialized SCD programs like those at UPMC [29, 30].

When considering the non‐SCA population (e.g., HbSC or HbSβ^+^), utilization rates were dramatically lower [18]. Although non‐SCA genotypes are generally associated with milder disease manifestations than SCA, these patients can still experience frequent VOCs and serious complications that compromise clinical outcomes [20]. Despite this clinical reality, only 8.0% of non‐SCA adults received HU within 1 year after meeting the NHLBI severity threshold for HU use. Our multivariable analysis confirmed this striking undertreatment pattern, revealing that SCA patients had 4.5 times higher odds of receiving HU early compared with non‐SCA patients. This finding aligns with a Nigerian study demonstrating low HU utilization among HbSC patients (the majority of non‐SCA genotypes) [31]. The disparity remained consistent across all sensitivity analyses in our study. To our knowledge, this represents the first US study exploring HU utilization specifically in non‐SCA patients who met the severity threshold recommended by the NHLBI clinical guidelines.

Beyond SCA genotypes, age emerged as another significant factor, with younger adults (18–21 years) having a higher likelihood of receiving HU early compared with older age groups across all sensitivity analyses, which aligns with previous studies conducted in Florida and Tennessee [12, 32]. This age‐related pattern likely reflects continuity from pediatric care, as patients transitioning from pediatric to adult care have higher HU prescription rates compared with older adults who face complex barriers spanning patient‐level (e.g., poor medication adherence), provider‐level (e.g., concerns about toxicity, reproductive effects, and comorbidities), and health care system‐level (e.g., lack of specialized care) factors [25, 33, 34, 35, 36, 37, 38, 39, 40]. Additionally, disease severity may influence HU prescribing decisions, with patients having severe complications being more likely to receive HU early, particularly those with pulmonary complications (which likely reflects ACS) [41, 42] which remained consistent across all our models and likely reflects physicians' tendency to prescribe HU in response to organ damage or life‐threatening complications [43, 44].

Our study identifies several factors contributing to current low HU utilization rates, underscoring the need for targeted interventions to address these barriers. Most importantly, we uncovered a critical treatment gap among non‐SCA patients who remain underrepresented in current HU treatment paradigms despite experiencing significant economic burden and adverse clinical outcomes. The low HU utilization rates in non‐SCA populations may reflect historical uncertainty about HU's benefits, as evidence from randomized controlled trials has been limited. However, a recent Nigerian trial demonstrated dramatic improvements among HbSC patients (who represent the majority of non‐SCA cases), with 62% reductions in VOC episodes and 58% decreases in hospitalizations, accompanied by mild hematologic toxicities across both pediatric and adult populations [45]. These findings suggest huge potential benefits from improved HU utilization in non‐SCA patients by preventing costly cycles of emergency care and hospitalizations. The economic implications are particularly striking, as non‐SCA adults experiencing frequent VOCs (≥ 3 episodes within 12 months) incur average health care costs of nearly $60,000 annually, according to Medicaid data [6]. Beyond health care costs, frequent VOCs also reduce health‐related quality of life and impact daily functioning on patients and families [46]. The treatment disparity becomes even more pronounced with age. Although adults with SCD experience more frequent and severe symptoms as the disease progresses, they are paradoxically least likely to receive HU [47]. Our study underscores the urgent need for US‐based trials to establish comprehensive safety and efficacy profiles for HU therapy in non‐SCA patients, which could provide the evidence base needed to enhance physician confidence and improve HU utilization across adult SCD populations.

Several important limitations must be considered. Our definition of early HU use captures only filled medication, and therefore cannot distinguish patients who were never prescribed HU from those who received a prescription but did not fill it. Furthermore, dispensing does not guarantee medication consumption or adherence. Our binary classification of early versus non‐early users and the 180 days of look‐back period may not capture complex treatment patterns and may misclassify patients who previously discontinued HU due to ineffectiveness or adverse effects as non‐early users, particularly older patients with longer disease histories. Misclassification bias may occur if patients meet qualifying criteria (≥ 3 VOCs) outside the UPMC system before transferring care or while receiving concurrent care elsewhere. This prevents accurate identification of their true guideline‐recommended HU initiation time and potentially results in misclassifying patient eligibility status and underestimating appropriate HU utilization rates. Additionally, our SCD identification algorithm was originally validated in pediatric populations using different EHR systems. Our cohort's lower SCA prevalence (45.5%) compared with typical reports (70%) [16, 48], suggests possible misclassification bias. However, this lower SCA prevalence likely attenuated rather than inflated our observed associations with early HU use, biasing our sample toward the reference (non‐SCA) group. This suggests that the true impact of SCA genotype on early HU use may be stronger than our results indicate.

## 5 Conclusion

This study reveals significant underutilization of HU therapy among adults with moderate to severe SCD for more than a decade after the 2014 NHLBI guidelines. Despite meeting guideline‐recommended severity thresholds, non‐SCA patients remain particularly undertreated (8.0% vs. 42.8% within 12 months for SCA patients). SCA genotype, younger age, and pulmonary complications are key predictors of early HU use. Given the substantial economic burden and reduced quality of life, these findings highlight critical treatment gaps requiring US‐based trials to establish HU safety and efficacy in non‐SCA genotypes [6, 46]. Future studies should also address age barriers to optimize HU use and improve outcomes across all SCD populations.

## Author Contributions

**Siang‐Hao Cheng:** conceptualization, investigation, writing – original draft, visualization, methodology, formal analysis, data curation, software. **Enrico M. Novelli:** writing – review and editing. **Hyeun Ah Kang:** writing – review and editing. **Terri V. Newman:** supervision, conceptualization, methodology, writing – review and editing. **Kangho Suh:** conceptualization, methodology, supervision, writing – review and editing.

## Funding

The authors have nothing to report.

## Conflicts of Interest

Terri V. Newman was employed by Novo Nordisk Inc. during the drafting of the final publication. Enrico M. Novelli reports being an advisory panel member and consulting work with Novo Nordisk. All other authors declare no conflicts of interest.

## Supporting information


**Table S1:** ICD‐9 and ICD‐10 Codes that define vaso‐occlusive crisis events.
**Table S2:** Algorithm to identify sickle cell anemia patients (HbSS/β^0^), adapted for the University of Pittsburgh Medical Center Electronic Health Records.
**Table S3:** Codes for inclusion/exclusion criteria and covariates.
**Table S4:** Temporal trends in early hydroxyurea utilization by index year and genotype.
**Table S5:** Baseline characteristics by early hydroxyurea use status: sensitivity analysis with 180‐day post‐index observation period.
**Table S6:** Sensitivity analyses for early hydroxyurea utilization.

## Data Availability

The data that support the findings of this study are available from the corresponding author upon reasonable request.
