# Supplementary material for: Genotype Differences and Hydroxyurea Utilization Among Adults With Moderate to Severe Sickle Cell Disease
Source: Pharmacotherapy. 2026 Jan 14;46(1):e70099. doi: 10.1002/phar.70099 (PMC12800872; doi:10.1002/phar.70099)
Supplement: Supplementary file 1 — Table S1: ICD‐9 and ICD‐10 Codes that define vaso‐occlusive crisis events. Table S2: Algorithm to identify sickle cell anemia patients (HbSS/β0), adapted for the University of Pittsburgh Medical Center Electronic Health Records. Table S3: Codes for inclusion/exclusion criteria and covariates. Table S4: Temporal trends in early hydroxyurea utilization by index year and genotype. Table S5: Baseline characteristics by early hydroxyurea use status: sensitivity analysis with 180‐day post‐index observation period. Table S6: Sensitivity analyses for early hydroxyurea utilization. [file PHAR-46-0-s001.docx]

**Supplementary Information**

**Supplementary Table 1. ICD-9 and ICD-10 Codes that Define Vaso-Occlusive Crisis Events**

|  | **Table Name** | **Variable** | **Data Values Used** |
| --- | --- | --- | --- |
| **STEP 1:** Identify encounters with sickle cell disease pain crises codes | DIAGNOSIS  DIAG_HOSP | DX_TYPE | 'ICD09', 'ICD10', 'ICD09CM', 'ICD10CM' |
|  | DIAGNOSIS  DIAG_HOSP | DX | - ICD-9: - Vaso-occlusive crisis: '282.42', '282.62', '282.64', '282.69' - Acute chest syndrome: '517.3' - Splenic sequestration: '289.52' - ICD-10: - HbSS disease with crisis: 'D57.0', 'D57.00', - Sickle-cell/Hb-C disease with crisis: 'D57.21', 'D57.211', 'D57.212', 'D57.219' - Sickle-cell thalassemia, unspecified, with crisis: 'D57.41', 'D57.411', 'D57.412', 'D57.419' - Sickle-cell thalassemia beta zero with crisis: 'D57.43', 'D57.431', 'D57.432', 'D57,439' - Sickle-cell thalassemia beta plus with crisis: 'D57.45', 'D57.451', 'D57.452', 'D57.459' - Other sickle-cell disorders with crisis: 'D57.81', 'D57.811', 'D57.812','D57.819' |
|  | DIAGNOSIS  DIAG_HOSP | DX_SOURCE | 'CERNER', 'EPIC', 'CERNER_CHP' |
| **STEP 2a:** Identify additional pain encounters using ICD codes | DIAGNOSIS  DIAG_HOSP | DX_TYPE | 'ICD09', 'ICD10', 'ICD09CM', 'ICD10CM' |
|  | DIAGNOSIS  DIAG_HOSP | DX | - Abdominal and pelvic pain - ICD-10: 'R10.0', 'R10.10', 'R10.11', 'R10.12', 'R10.13', 'R10.2', 'R10.30', 'R10.31', 'R10.32', 'R10.33', 'R10.84', 'R10.9' - ICD-9: '789.00', '789.01', '789.02', '789.03', '789.04', '789.05’, 789.06', '789.07', '789.09' - Pain in chest - ICD-10: 'R07.2', 'R07.89', 'R07.9', - ICD-9: '786.50', '786.51', '786.59' - Pain in joint - ICD-10: 'M25.50', 'M25.511', 'M25.512', 'M25.519', 'M25.521', 'M25.522', 'M25.531', 'M25.532', 'M25.551', 'M25.552', 'M25.559', 'M25.561', 'M25.562', 'M25.569', 'M25.571', 'M25.572', 'M25.579' - ICD-9: '719.40', '719.41', '719.42', '719.43', '719.44', '719.45', '719.46', '719.47', '719.48', '719.49' - Pain in limb, hand, foot, fingers and toes - ICD-10: 'M79.601', 'M79.602', 'M79.603', 'M79.604', 'M79.605', 'M79.606', 'M79.609', 'M79.621', 'M79.622', 'M79.629', 'M79.631', 'M79.632', 'M79.639', 'M79.641', 'M79.642', 'M79.643', 'M79.644', 'M79.645', 'M79.646', 'M79.651', 'M79.652', 'M79.659', 'M79.661', 'M79.662', 'M79.669', 'M79.671', 'M79.672', 'M79.673', 'M79.674', 'M79.675', 'M79.676' - ICD-9: '729.5' - Cervicalgia, Low back pain, Pain in thoracic spine - ICD-10: 'M54.2', 'M54.5', 'M54.6' - ICD-9: '723.1', '724.1', '724.2', '724.5' - Other Priapism, or unspecified: - ICD-10: 'N48.30', 'N48.32', 'N48.39' - ICD-9: '607.3' |
|  | DIAGNOSIS  DIAG_HOSP | DX_SOURCE | 'CERNER', 'EPIC', 'CERNER_CHP' |

**Abbreviation:** ICD-9=International Classification of Diseases, Ninth Revision; ICD-10= International Classification of Diseases, Tenth Revision

**Supplementary Table 2. Algorithm to Identify Sickle Cell Anemia Patients (HbSS/β⁰), Adapted for the University of Pittsburgh Medical Center Electronic Health Records**

|  | **Table Name** | **Variable** | **Data Values Used** |
| --- | --- | --- | --- |
| **STEP 1a:** Determine SCD patients' genotype based on the most common ICD code  **STEP 1b:** For SCD patients with 'non-specific' genotype classification, determine genotype based on the second most common ICD code | DIAGNOSIS  DIAG_HOSP | DX_TYPE  DIAGNOSIS_TYPE | 'ICD09', 'ICD10', 'ICD09CM', 'ICD10CM', |
|  | DIAGNOSIS  DIAG_HOSP | DX _CODE | - ICD-9:   '282.41', '282.42', '282.61', '282.62', '282.63', '282.64', '282 .68', '282.69'   - ICD-10:   'D57.0', 'D57.01', 'D57.02', 'D57.1', 'D57.2', 'D57.20', 'D57.21', 'D57.211', 'D57.212', 'D57.219','D57.4', 'D57.40', 'D57.41', 'D57.411', 'D57.412', 'D57.419', 'D57.8', 'D57.80', 'D57.81', 'D57.811', 'D57.812', 'D57.819'  Excluding : '282.5', 'D57.3' |
|  | DIAGNOSIS  DIAG_HOSP | DX_SOURCE | 'CERNER', 'EPIC', 'CERNER_CHP' |
| **STEP 2:** If the patient's genotype remains undetermined and the patient has had HbS ≥80% at any time point, along with no or missing HbA1 and HbA2, then classify as SCA. If the patient has any results for HbC or other hemoglobin variants, then classify as “other sickle cell disease”. | LAB_RESULTS | COMPONENT_NAME | 'HEMOGLOBIN A1',' HEMOGLOBIN A2',' HEMOGLOBIN C', 'HEMOGLOBIN E', 'Hemoglobin S', 'Rapid hemoglobin S' |
|  | LAB_RESULTS | ORD_VALUE |  |
| **STEP 3:** If the patient had a Transcranial Doppler ultrasound order, then classify as SCA. | PROC_PREFORMED | CPT_CODE | '93886', '93888' |
| COMPONENT_NAME | | Description | |
| HEMOGLOBIN A1 | | Hemoglobin A1/Hemoglobin total in Blood | |
| HEMOGLOBIN A2 | | Hemoglobin A2/Hemoglobin total in Blood | |
| HEMOGLOBIN C | | Hemoglobin C/Hemoglobin total in Blood | |
| HEMOGLOBIN E | | Hemoglobin E/Hemoglobin total in Blood | |
| Hemoglobin S | | Hemoglobin S/Hemoglobin total in Blood | |
| Rapid hemoglobin S | | Hemoglobin S/Hemoglobin total in Blood by HPLC | |

**Abbreviation:** ICD-9=International Classification of Diseases, Ninth Revision; ICD-10= International Classification of Diseases, Tenth Revision; SCD=Sickle Cell Disease; SCA=Sickle Cell Anemia; HbS=Hemoglobin S; HbA1=Hemoglobin A1; HbA2=Hemoglobin A2; HbC=Hemoglobin C

**Supplementary Table 3. Codes for Inclusion/Exclusion Criteria and Covariates**

| **Variables** | **CPT-4¹/ ICD-10-PCS⁶/ HCPCS⁷** | **ICD-9²/ ICD-9-CM³** | **ICD-10⁴/ ICD-10-CM⁵** |
| --- | --- | --- | --- |
| **Inclusion/Exclusion Criteria** | | | |
| Sickle Cell Disease |  | 282.41, 282.42, 282.6, 282.6x | D57.0, D57.01, D57.02, D57.1, D57.2, D57.20, D57.21, D57.211, D57.212, D57.219, D57.4, D57.40, D57.41, D57.411, D57.412, D57.419, D57.8, D57.80, D57.81, D57.811, D57.812, D57.819 |
| Allogeneic HSCT**⁸**/HSCI**⁹** | **CPT-4:** 38240 |  |  |
| Malignancies that may require HU treatment |  | **Melanoma**  172, 172.x  **Resistant chronic myeloid leukemia**  205.10  **Locally advanced squamous cell carcinomas of the head and neck, (excluding lips) in combination with concurrent chemoradiation**  195.0  **Recurrent, metastatic or inoperable carcinoma of the ovary**  95.0 | **Melanoma**  C43, C43.x, C43.xx  **Resistant chronic myeloid leukemia**  C92.10, C92.12, C92.20, C92.22  **Locally advanced squamous cell carcinomas of the head and neck, (excluding lips) in combination with concurrent chemoradiation**  C76.0  **Recurrent, metastatic or inoperable carcinoma of the ovary**  C56.1, C56.2, C56.9 |
| **Covariates** | | | |
| Stroke (ischemic/hemorrhagic)/TIA**¹⁰** |  | 433.x1, 434.x1, 430, 431, 432, 435.x | I63 (all), I60 (all), I61.x, I62(all), G45 (all), |
| Acute Chest Syndrome |  | 517.3 | D57.01, D57.211, D57.411, D57.431, D57.451, D57.811 |
| Splenic sequestration |  | 289.52 | D57.02, D57.212, D57.412, D57.432, D57.452, D57.812, |
| Multi-Organ Failure |  | 995.92, 995.94 | R65.11, R65.20, R65.21 |
| Avascular necrosis |  | 733.4x | M87.3x, M87.3xx, M87.8, M87.8x, M87.8xx, M87.9 |
| Pulmonary complications (pneumonia, URTI**¹¹**, pulmonary embolism, pulmonary HTN**¹²**) |  | 481, 465.x, 415.12, 415,13, 415.19, 416.2, 416.8 | J13, J00, J01.xx, J02.x, J03.x, J03.xx, J04.x, J04.xx, J05.x, J05.xx, J06.x, I26.0x, I26.9x, I27.82, I27.2x, I29.29, I27.81, I27.82, I27.89, I27.9 |
| Renal disease (CKD**¹³**, renal failure (acute or chronic), acute and chronic glomerulonephritis) |  | 593.9, 584.x, 585.x, 586, 587, 588.x, 588.xx, 580.x, 580.xx, 581.x, 581.xx, 582.x, 582.xx, 582.xx. 583.x, 583.xx | N00.x, N01.x, N02.x, N03.x, N04.x, N05.x, N06.x, N07.x, N08, N18.9, N17.x, N18.x, N18.xx, N19, N25.x, N25.xx, N26.9 |
| Thrombosis |  | 452, 453.x, 453.xx | I81, I82.x, I82.xx, I82.xxx |
| Leg ulcers |  | 454.0, 454.2, 707.1x | I83.0x, I83.0xx, I83.2, I83.2x, I83.2xx, L97.xxx |
| COPD**¹⁴**/asthma |  | 402.01, 402.11, 402.91, 404.01, 404.03, 404.11, 404.13, 404.91, 404.93, 490, 493.0x, 493.1x, 493.2x, 493.9x, 491.x, 491.xx, 492.x, | J45.2, J45.2x, J45.3x, J45.4x, J45.5, J45.5x, J45.90, J45.90x, J40, J41.x, J42, J43.x, J44.x |
| HF**¹⁵** |  | 428 (all), 398.91, 402.01, 402.11, 402.91, 404.01, 404.03, 404.11, 404.13, 404.91, 404.93 | I09.81, I11.0, I13.0, I13.2, I50.1, I50.2x, I50.3x, I50.4x, I50.9 |
| CAD**¹⁶** |  | 414.0x, 414.2, 414.2, 414.3, 414.4 | I25.10, I25.11x |
| Diabetes |  | 250.xx, 357.2, 366.41, 362.0x | E11 (all), E10 (all) |
| HTN |  | 401.x | I10 |
| Hyperlipidemia |  | 272.x, 272.1x, 272.2x, 272.4x, | E78.00, E78.01, E78.1, E78.2, E78.3, E78.41, E78.49, E78.5 |
| Anxiety |  | 300.0x | F41.x |
| Depression |  | 296.2x, 296.3x, 311 | F32.x, F33 (all) |
| Bipolar disorder |  | 296.0x,296.1x, 296.7, 296.4x, 296.5x, 296.6x, 296.7, 296.8x, 296.9x | F31.0, F31.1x, F31.2, F31.3x, F31.4, F31.5, F31.6x, F31.7x, F31.8x, F31.9 |
| Schizophrenia |  | 295 (all) | F20 (all), F25 (all) |
| Neoplasms |  | 140.x-172.x, 174.x-195.8, 200.x-208.x, 238.6 | C00-C41, C45-D49 |
| Simple Transfusion | **CPT-4:**  09882, 36430, 36440, 86890, 86999, 86927, 86930, 86931, 86932  **ICD-10-PCS:**  30233H1, 30243H1, 0233N1, 30233P1, 30243N1, 30243P1  **HCPCS:**  S3906, P9016, P9010, P9021, P9022, P9038, P9039, P9017, P9040 | 99.03, 99.04 |  |
| Exchange Transfusion | **CPT-4:**  09883, 36450, 36455, 36512, 36456  **ICD-10-PCS:**  6A550Z0 | 99.01, 99.73 |  |

***Abbreviations:*** *¹CPT-4: Current Procedural Terminology, 4th Edition; ²ICD-9: International Classification of Diseases, 9th Revision; ³ICD-9-CM: International Classification of Diseases, 9th Revision, Clinical Modification; ⁴ICD-10: International Classification of Diseases, 10th Revision; ⁵ICD-10-CM: International Classification of Diseases, 10th Revision, Clinical Modification; ⁶ICD-10-PCS: International Classification of Diseases, 10th Revision, Procedure Coding System; ⁷HCPCS: Healthcare Common Procedure Coding System; ⁸HSCT: Hematopoietic Stem Cell Transplantation; ⁹HSCI: Hematopoietic Stem Cell Infusion; ¹⁰TIA: Transient Ischemic Attack; ¹¹URTI: Upper Respiratory Tract Infection; ¹²HTN: Hypertension; ¹³CKD: Chronic Kidney Disease; ¹⁴COPD: Chronic Obstructive Pulmonary Disease; ¹⁵HF: Heart Failure; ¹⁶CAD: Coronary Artery Disease*

**Supplementary Table 4. Temporal Trends in Early Hydroxyurea Utilization by Index Year and Genotype**

| **Index Year** | **Total Patients** | **Early HU^1^ Use Rate (Overall)** | **SCA^2^ Patients** | **Early HU Use Rate (SCA)** | **Non-SCA Patients** | **Early HU Use Rate (non-SCA)** |
| --- | --- | --- | --- | --- | --- | --- |
|  | **N** | **n (%)** | **N** | **n (%)** | **N** | **n (%)** |
| **2014** | 77 | 20 (25.97%) | 45 | 19 (42.22%) | 32 | 1(3.13%) |
| **2015** | 77 | 13 (16.88%) | 36 | 11 (50.56%) | 41 | 2 (4.88%) |
| **2016** | 49 | 8 (16.33%) | 24 | 8 (33.33%) | 25 | 0 (0%) |
| **2017** | 41 | 5 (12.2%) | 16 | 4 (25%) | 25 | 1 (4%) |
| **2018** | 38 | 6 (15.79%) | 16 | 5 (31.25%) | 22 | 1 (4.55%) |
| **2019** | 35 | 3 (8.57%) | 12 | 0 (0%) | 23 | 3 (13.04%) |
| **2020** | 39 | 8 (20.51%) | 14 | 6 (42.86%) | 25 | 2 (8%) |
| **2021** | 32 | 9 (28.13%) | 19 | 7 (36.84%) | 13 | 2 (15.38%) |
| **2022** | 10 | 3 (30%) | 3 | 2 (66.67%) | 7 | 1 (14.29%) |
| **2023** | 13 | 5 (38.46%) | 2 | 2 (100%) | 11 | 3 (27.07%) |

***Abbreviations:*** *^1^HU: Hydroxyurea; ^2^SCA: Sickle cell anemia*

*Index year refers to the calendar year in which each patient had their index date. SCA includes patients with HbSS and HbSβ⁰-thalassemia genotypes. Non-SCA includes patients with HbSC, HbSβ⁺ thalassemia, and other sickle cell disease variants. Percentages represent the proportion of patients who used HU within 90 days post-index; n indicates the number of early HU users. Total patients (N) represent all patients with an index date in that calendar year who met the severity threshold for HU recommendation. Sample sizes vary considerably by year. Smaller cohorts in later years reflect both the requirement for adequate follow-up time to assess HU utilization and our study design, which assigned each patient's earliest qualifying episode (≥3 VOCs within 12 months) as their index date.*

**Supplementary Table 5. Baseline characteristics by early hydroxyurea use status: sensitivity analysis with 180-day post-index observation period**

| **Characteristic** | | | | |  |
| --- | --- | --- | --- | --- | --- |
|  | **Objective 2** | | | | |
|  | **All (N=411)** | **Early HU users (N=89)** | **Non-Early HU users (N=322)** | **P-value** | |
|  | **N (%)** | | |  | |
| **Age at index date** | | | | | |
| Mean ± SD | 42.36±17.92 | 30.54±11.08 | 45.63±18.01 | **<0.0001^a^** | |
| Median (Q1, Q3) | 40.30 (26.10,56.00) | 26.80 (22.50, 38.10) | 45.65 (29.50,58.10) |  |  |
| **Age groups** | | | | | |
| 18-21 | 48 (11.68) | 20 (22.47) | 28 (8.70) | **<0.0001^b^** | |
| 22-30 | 85 (20.68) | 30 (33.71) | 55 (17.08) |  |  |
| 31-40 | 72 (17.52) | 20 (22.47) | 52 (16.15) |  |  |
| 41+ | 206 (50.12) | 19 (21.35) | 187 (58.07) |  |  |
| **Sex** | | | | | |
| Females | 252 (61.31) | 45 (52.56) | 207 (64.29) | **0.02^b^** | |
| Males | 159 (38.69) | 44 (49.44) | 115 (35.71) |  |  |
| **SCA** | 187 (45.50) | 64 (80.00) | 123 (37.16) | **<0.0001^b^** | |
| **History of Transfusion** | 24 (5.84) | 10 (11.24) | 14 (4.35) | **0.01^b^** | |
| **Opioid Use** | 153 (37.23) | 39 (43.82) | 114 (35.40) | 0.15**^b^** | |
| **Prior HU use** | 73 (17.76) | 67 (75.28) | 6 (1.86) | **<0.0001** | |
| **VOC episode count** | | | | | |
| Mean ± SD | 1.71±1.69 | 2.16±2.70 | 1.59±1.26 | 0.51^b^ | |
| Median (Q1, Q3) | 2.00 (1.00,2.00) | 2.00 (1.00,2.00) | 2.00 (1.00,2.00) |  |  |
| **Stroke** | 16 (3.89) | 3 (3.37) | 13 (4.04) | 1**^c^** | |
| **AVN** | 7 (1.70) | 5 (5.62) | 2 (0.62) | <0.01**^b^** | |
| **Renal disease** | 26 (6.33) | 7 (7.87) | 19 (5.90) | **0.50^b^** | |
| **Pulmonary complications** | 64 (15.57) | 25 (28.09) | 39 (12.11) | **<0.01^b^** | |
| **Thrombosis** | 16 (3.89) | 3 (3.37) | 13 (4.04) | 1**^c^** | |
| **Leg Ulcers** | 8 (1.95) | 5 (5.62) | 3 (0.93) | **0.01^c^** | |
| **COPD / Asthma** | 78 (18.98) | 17 (19.10) | 61 (18.94) | 0.97^b^ | |
| **HF / CAD** | 27 (6.57) | 4 (4.49) | 23 (7.14) | 0.37^b^ | |
| **DM / HTN / HLD** | 116 (28.22) | 9 (10.11) | 107 (33.23) | **<0.001**^b^ | |
| **Anxiety/ Depression** | 98 (23.84) | 13 (14.61) | 85 (26.40) | **0.02**^b^ | |

***Test performed:*** ***^a^*** *Mann-Whitney U test, ^b^Chi-square test, ^c^Fisher's exact test****No patients experienced multiorgan failure during the study period***
***Abbreviations:*** *SCD = Sickle Cell Disease; SCA = Sickle Cell Anemia; HU = Hydroxyurea; VOC = Vaso-occlusive crisis; TIA = Transient ischemic attack; CKD = Chronic kidney disease; URTI = Upper respiratory tract infection; PE = Pulmonary embolism; PH = Pulmonary hypertension; AVN=Avascular necrosis****;*** *COPD = Chronic obstructive pulmonary disease; HF = Heart failure; CAD = Coronary artery disease; DM = Diabetes mellitus; HTN = Hypertension; HLD = Hyperlipidemia*

**Supplementary Table 6. Sensitivity Analyses for Early Hydroxyurea Utilization**

|  | **Full Model** | | | | **Sickle Cell Anemia only** | | | | **180-day treatment window** | | | |  |
| --- | --- | --- | --- | --- | --- | --- | --- | --- | --- | --- | --- | --- | --- |
|  | OR | LCI | UCI | p-value | OR | LCI | UCI | p-value | OR | LCI | UCI | p-value |  |
| **Age 22-30 vs 18-21** | 0.85 | 0.30 | 2.47 | 0.14 | 0.77 | 0.29 | 2.04 | **0.03** | 0.82 | 0.36 | 1.88 | **0.03** |  |
| **Age 31-40 vs 18-21** | 0.34 | 0.11 | 1.07 | 0.16 | 0.45 | 0.16 | 1.24 | 0.76 | 0.50 | 0.21 | 1.19 | 0.95 |  |
| **Age 40+ vs 18-21** | 0.31 | 0.10 | 0.99 | 0.10 | 0.08 | 0.03 | 0.25 | **<.0001** | 0.14 | 0.06 | 0.34 | **<.0001** |  |
| **Male vs Female** | 1.18 | 0.56 | 2.49 | 0.67 | 1.02 | 0.50 | 2.08 | 0.96 | 1.30 | 0.73 | 2.31 | 0.37 |  |
| **SCA vs non-SCA** | 2.00 | 0.90 | 4.44 | 0.09 |  | | | | 5.96 | 3.15 | 11.27 | **<.0001** |  |
| **History of Transfusion** | 1.02 | 0.27 | 3.91 | 0.98 | 1.15 | 0.37 | 3.54 | 0.81 | 0.96 | 0.34 | 2.67 | 0.94 |  |
| **Opioid Use** | 0.64 | 0.27 | 1.51 | 0.31 | 2.16 | 1.00 | 4.68 | 0.05 | 2.19 | 1.20 | 4.01 | **0.01** |  |
| **Prior HU use** | 41.60 | 17.19 | 100.68 | **<.0001** |  | | | |  | | | |  |
| **VOC episode counts** | 0.88 | 0.73 | 1.07 | 0.20 | 1.06 | 0.88 | 1.28 | 0.54 | 1.02 | 0.87 | 1.19 | 0.82 |  |
| **Renal Disease** | 3.88 | 0.93 | 16.25 | 0.06 |  | | | |  | | | |  |
| **Pulmonary Complications** | 1.65 | 0.63 | 4.33 | 0.31 | 3.03 | 1.28 | 7.17 | **0.01** | 2.45 | 1.22 | 4.90 | **0.01** |  |
| **COPD/Asthma** | 1.44 | 0.57 | 3.61 | 0.44 | 1.28 | 0.49 | 3.37 | 0.61 | 0.96 | 0.45 | 2.03 | 0.91 |  |
| **HF/CAD** | 1.33 | 0.24 | 7.41 | 0.74 |  | | | |  | | | |  |
| **DT/HTN/HL** | 0.28 | 0.08 | 0.96 | **0.04** |  |  |  |  |  |  |  |  |  |
| **Anxiety/**  **Depression** | 0.76 | 0.29 | 1.99 | 0.57 | 0.56 | 0.18 | 1.76 | 0.32 | 0.73 | 0.34 | 1.60 | 0.43 |  |
| **AUC** | 0.93 | | | | 0.77 | | | | 0.83 | | | |  |

***Abbreviations:*** *OR = Odds Ratio; LCI = Lower Confidence Interval; UCI = Upper Confidence Interval; SCA = Sickle Cell Anemia; HU = Hydroxyurea; VOC = Vaso-occlusive crisis; COPD = Chronic obstructive pulmonary disease; HF = Heart failure; CAD = Coronary Artery Disease; DT = Diabetes Type; HTN = Hypertension; HLD = Hyperlipidemia.*
